# High penetrances of *BRCA1 *and *BRCA2 *mutations confirmed in a prospective series

**DOI:** 10.1186/1897-4287-8-2

**Published:** 2010-01-19

**Authors:** Pål Møller, Lovise Mæhle, Lars F Engebretsen, Trond Ludvigsen, Christoffer Jonsrud, Jaran Apold, Anita Vabø, Neal Clark

**Affiliations:** 1 Section of Cancer Genetics, The Norwegian Radium Hospital, Oslo University Hospital, N-0310 Oslo, Norway; 2 Regional Competence Center for Familial Cancer, Helse Vest, Haukeland University Hospital, N-5021 Bergen, Norway; 3 Section of Medical Genetics, Department of Pathology and Medical Genetics, St. Olavs Hospital, N-7006 Trondheim, Norway; 4 University Hospital of North Norway, Division of Child and Adolescent Health, Department of Medical Genetics, N-9038 Tromsø, Norway

## Abstract

Penetrances of *BRCA1 *and *BRCA2 *mutations have been derived from retrospective studies, implying the possibility of ascertainment biases to influence the results.

We have followed women at risk for breast and/or ovarian cancer for two decades, and report the prospectively observed age-related annual incidence rates to contract breast or ovarian cancer for women with deleterious *BRCA1 *or *BRCA2 *mutations based on 4830 observation years. Patients were grouped according to mutation, age and having/not having had previous cancer.

In women not having had previous cancer and aged 40-59 years, the annual incidence rate to contract breast or ovarian cancer in those having the most frequent *BRCA1 *founder mutations was 4.0%, for women in this age group and with less frequent *BRCA1 *mutations annual incidence rate was 5.9%, and for women with *BRCA2 *mutations 3.5%.

The observed figures may be used for genetic counseling of healthy mutation carriers in the respective age groups. The results may indicate that less frequent *BRCA1 *mutations have higher penetrances than *BRCA1 *founder mutations.

## Introduction

Mutations in the two genes *BRCA1 *and *BRCA2 *may cause breast or ovarian cancer. Estimates on penetrances have been based on retrospective studies and have arrived at diverging results.

Retrospective reports include the possibility of presenting the selection criteria as results [1]. Some studies tested one affected proband and calculated on number of affected close relatives [2,3]. In such studies, prevalences of close relatives to be mutation carriers, as well as age of onset of disease in the relatives, is related to ascertainment of the proband (age, complete or incomplete ascertainment), and the reported results reflect assumptions on these factors. Others reported findings based on mutation testing in extended families to avoid some of these confounders and arrived at higher penetrance estimates [4-7]. Reports including prospective series are limited [8-10].

The suggestion that genetic modifying factors of penetrance should underly the conflicting results has not been confirmed, but the possibility of three modifiers inferring hazard ratio (HR) of 1.1 to 1.3 in *BRCA2 *mutation carriers, and one locus with HR 1.1 for *BRCA1 *remain. The three loci are outside linkage distance for both *BRCA1 *and *BRCA2 *and will combined with the HRs reported have marginal impact on total risk estimates for relatives [11]. Discussing the theoretical concepts of modifiers of penetrances and expressions in depth, as well as the pitfalls in examining for such factors, are outside the scope of this reports.

We have suggested that all incident cases of breast or ovarian cancer in Norway should be offered testing for the Norwegian deleterious *BRCA1 *and BRCA2 mutations. While considering our suggestion, the Norwegian Government asked for documentation of the penetrances of the mutations in question. We decided to analyze our prospective series to arrive at prospectively, empirically observed annual incidence rates to answer the question, and report our findings here. The main goal was to describe annual incidence rate in those who had had no cancer before inclusion and who had no cancer demonstrated at first (prevalence) round.

## Materials and methods

We have subjected healthy women at risk for breast or ovarian cancer by family history to prospective follow-up for two decades. Details on ascertainment methods have been previously published [12-17]. Genetic testing was facilitated by the demonstration of Norwegian founder mutations and rapid and cheap tests to demonstrate them. BRCA founder mutations is not specific for Norway, but the mutations are different in different ethnic groups [6,18]. Close to all families assumed by family history to have inherited breast or ovarian cancer have been tested for at least the 10 most frequent Norwegian mutations (for review, test panel and geographical distribution of the Norwegian founder mutations, see Møller et al. [16]).

The families were ascertained according to preset criteria as previously published [7]. We examined at least one affected per family with sequencing and MLPA, if available. If no affected was available, we have in many families sequenced obligate male carriers or young daughters.

As mentioned above, most BRCA mutations demonstrated in Norway have passed through the bottleneck caused by the Bubonic plagues 25 generations ago, and the population parts have lived separately until a few generations ago [16]. The consequence is that most mutations still are located to one single geographical area, and the families themselves are aware of this and give a precise description if asked. We have over two decades classified the families according to both geographical origin and present address of current members. Our electronic medical files include zip-codes for all women either with breast or ovarian cancer or at risk according to family history, and were used to identify families for geographical locations. Whenever a mutation not included in the general test panel was described, the family was extended as far as possible, the likely geographical origin(s) of the mutation determined, and all families connected to that area were tested for that mutation.

We included mutations causing direct stop, frameshift or large insertions/deletions and splice defects. It is agreed that such mutations are considered deleterious (see BIC: http://research.nhgri.nih.gov/bic/). To avoid discussions on validity of results, we excluded missense mutations in the finger domains considered deleterious by some [2], all forms of mutations in the last coding exon in *BRCA2*, and all intron variations outside +/- two bases from the exons considered to be splice donor or receptor sites. We did, however, include the BRCA c.1A>G mutation as it is locally frequent in one area and obviously follows the disease in all families affected (to be reported separately). A list of the deleterious mutations included in the present report, is posted on our web-site http://www.inherited-cancer.com.

Whenever a mutation was found, predictive testing was offered to the family members pending consent from the proband to do so. Compliance to predictive testing has been demonstrated to be high [19]. Most families are expanded far beyond any selection criteria, many of them to distant relatives often more than 5 meioses apart from the index person. In one family, a *de-novo BRCA2 *c.8090_8115del16 mutation was demonstrated (both parents without mutation, paternity confirmed by DNA testing).

Mutation testing was performed under national legislation including genetic counseling and written informed consent for each single patient. No named information was exported from the medical files, no research registry including patient identifications was erected. The medical database and application was constructed by Oracle 10 g^© ^and Delphi 2007^© ^and the data for this report extracted by TOAD^© ^by PM and NC.

All healthy women with a deleterious *BRCA1 *or *BRCA2 *mutation were offered annual follow-up from age 25 years on, aiming at early diagnosis and treatment. The same offer was given women with past or present cancer, if they were assumed to benefit.

The patients have been followed with annual examinations [12-17], and some have opted for risk-reducing surgery [17,20]. Observation time was calculated as time between first and last recorded results. All cases of breast or ovarian cancers in the observation period were recorded as events irrespective of detection method, including interval cancers. No other cancer or any other disease was scored as event. Each woman was counted once only for having or not having breast or ovarian cancer prior to inclusion. Each woman was scored once only for cancer at first examination irrespective of how many tumours possibly detected. Each woman was scored once only for having or not having demonstrated breast or ovarian cancer at follow-up. In theory, one woman may have been scored for having cancer in all three groups mentioned, but not more than once in any group.

All data from the reporting centres including March 2009 are reported. Founder *BRCA1 *mutation series from Bergen was incomplete, and all *BRCA1 *founder mutation carriers from Bergen were excluded.

Observation time was censored at first demonstrated breast or ovarian cancer at follow-up. Observation time was censored at both bilateral risk-reducing mastectomy and oophorectomy. No correction was done for those having had only the one or the other risk-reducing intervention. As previously reported, ovarian cancer may have some time in preclinical detectable stage, and we did diagnose some through risk-reducing oophorectomy [17]. Censoring the study at that time, might have given false high penetrance estimates. The way we did it, implies the risk of arriving at too low penetrance estimates, which we decided to prefer.

Age related annual incidence rates were calculated as number of women who contracted one or more cancer divided by numbers of observation years of women having the ages at first control as specified in Table 1 and Table 2.

**Table 1:** Results of follow-up: Women without cancer before and at first (prevalence) control.

*Mutation group*	*Age group*	*n*	*Observation* *years*	*Cases with prospective cancer*	*Mean observation time*	*Annual incidence rate*
B1F	<40	195	1282	27	6.6	**2.1%**
	40-59	121	749	30	6.2	**4.0%**
	60+	19	121	2	6.4	**1.7%**
	**SUM**	335	2152	59	**6.4**	**2.7%**
B1NF	<40	119	721	18	6.1	**2.5%**
	40-59	62	320	19	5.2	**5.9%**
	60+	10	60	3	6.0	**5.0%**
	**SUM**	191	1101	40	**5.8**	**3.6%**
B2	<40	64	347	5	5.4	**1.4%**
	40-59	51	258	9	5.1	**3.5%**
	60+	8	29	2	3.6	**6.9%**
	**SUM**	123	634	16	**5.2**	**2.5%**
***ALL***	***SUM***	***649***	***3887***	***115***	***6.0***	***3.0%***

B1F: *BRCA1 *founder mutations (1675delA, 1135insA, 3347delAG, 816delGT)

B1NF: All other *BRCA1 *mutations

B2: *BRCA2 *mutations

**Table 2:** Results of follow-up: Women with cancer before or at first (prevalence) control.

*Mutation group*	*Age group*	*n*	*Observation* *years*	*Cases with prospective cancer*	*Mean observation time*	*Annual incidence rate*
B1F	<40	20	78	5	3.9	**6.4%**
	40-59	64	299	7	4.7	**2.3%**
	60+	16	55	3	3.4	**5.5%**
	**SUM**	100	432	15	**4.3**	**3.5%**
B1NF	<40	21	111	2	5.3	**1.8%**
	40-59	38	173	3	4.6	**1.7%**
	60+	15	42	4	2.8	**9.5%**
	**SUM**	74	326	9	**4.4**	**2.8%**
B2	<40	4	19	2	4.8	**10.5%**
	40-59	28	119	3	4.3	**2.5%**
	60+	15	47	3	3.1	**6.4%**
	**SUM**	47	185	8	**3.9**	**4.3%**
***ALL***	***SUM***	***221***	***943***	***32***	***4.3***	***3.4%***

B1F: *BRCA1 *founder mutations (1675delA, 1135insA, 3347delAG, 816delGT)

B1NF: All other *BRCA1 *mutations

B2: *BRCA2 *mutations

Confidence intervals of means were considered by assuming Poisson distributions, groups were compared two-by-two by Fishers' exact p.

## Results

All together 1055 women were identified as having a deleterious *BRCA1*/2 mutation and examined once or more. At first (prevalence) round, 54 among them were diagnosed as having breast and/or ovarian cancer, arriving at a prevalence of 5.1%.

Among these, 870 were examined twice or more. Through 4830 follow-up years 147 among them were diagnosed as having breast and/or ovarian cancer, arriving at an overall annual incidence rate irrespective of age to be 3.0%.

These 870 were fractionated into three groups: a) the four *BRCA1 *founder mutations for which we have previously reported retrospective cumulative incidence rates, b) other *BRCA1 *mutations, and c) *BRCA2 *mutations. These three groups were split into two groups each: Those with no cancer prior to inclusion as well as with no cancer at first control, and those who did have a cancer prior to inclusion or demonstrated cancer at first control. The overall annual incidence rates irrespective of age in those who had no prior or prevalent cancer, were 2.7% for *BRCA1 *founder mutations, 3.6% for less frequent *BRCA1 *mutations, and 2.5% for *BRCA2 *mutations.

Then we fractionated each of the six groups defined above, into three groups each: those aged 25-39 years at inclusion, those aged 40-59 years at inclusion, and those aged 60 years or more at inclusion. The results for women without a previous cancer and no cancer at prevalence round are detailed in Table 1. In the age group 40-59 years at inclusion, annual incidence rates were 4.0% for *BRCA1 *founder mutations, 5.9% for less frequent *BRCA1 *mutations, and 3.5% for *BRCA2 *mutations. For all age groups, the annual incidence rates for women with founder mutations were less than for women with less frequent *BRCA1 *mutations. None of the differences between the three mutation groups were statistically significant.

The corresponding details for patients having had breast or ovarian cancer before inclusion, or at first control, are given in Table 2.

## Discussion

This prospective report confirms the previously reported penetrance estimates for the four most frequent *BRCA1 *mutations in Norway. As the healthy mutation carriers reported here are the next generation in the families previously reported, the finding was that disease continued to occur prospectively as previously reported retrospectively in these families.

The point estimates for annual incidence rates for the less frequent *BRCA1 *mutation carriers were higher than for those having a *BRCA1 *founder mutation. This may be a methodological artifact, as only one third of the less frequent BRCA1 mutations carried by the prospective cases, were included in the test panel applied to all families (data not given in results). However, as described in methods above we have over years searched for all the rare mutations in the geographical areas they are connected to, and have described a number of delineated areas with local founder mutations not present in the other population parts. The majority of families carrying rare mutations were not detected through sequencing a prospectively demonstrated cancer case: In the files at The Norwegian Radium Hospital which was available for detailed analysis, 547 women in 127 different kindreds were identified to have rare BRCA1 mutations, while the number of prospectively detected cancers in women with no cancer prior to or at first control and with rare BRCA1 mutations not included in the test panel used for all families, was 24. The influence of a selection error remains, but the probability that the selection problem had a major effect on the results may be limited.

An alternative speculation may be that there may have been an element of selection: The rare mutations may be rare because they have reduced fitness. If so, previous reports based on the most frequent mutations may be underestimates if applied to the less frequent mutations. If true, this may explain some of the discrepancies in the literature discussed above. The resources needed to clarify this, would imply full-scale sequencing in numbers which were not available to us and still not is. An answer may await new technology making sequencing of large numbers awailable and affordable.

We here report incidence rate as number of cases affected among the patients exposed during a given period of time [21], and grouped the patients according to age at first examination. As seen in the tables, the average follow-up period was about 6 years, meaning that the observed annual incidence rates reflected the risk per year of becoming affected the next 6 years for healthy women in the given age groups.

Retrospective reports are most often constructed differently: they report cumulative incidence rate by age, starting at birth. We did so in our previous retrospective reports [4,7]. For comparison with the current results, we recalculated our previous figures to reflect age related annual incidences among those who had experienced no cancer before reaching the various age groups. Doing so, the annual incidence rates in the retrospective series were 1.0%, 3.0%, 3.5% and 2.5% for the age groups 30-39, 40-49, 50-59 and 60-69 years, respectively (see Heimdal et al [7], table three, coloumn C for the data used for calculations). This is close to what we prospectively observed in the present series. The younger group <40 years in the present series had an average age at inclusion of 32 years and was followed for an average of 6 years, meaning that the mean age was about 38 years and the annual incidence rate was expected to be higher than derived from the retrospective series for the age group 30-39. The lower annual incidence rate observed in the older group 60+ years may reflect a diminishing annual incidence rate in older ages. All variations discussed here were, however, statistically insignificant and may have been caused by random variation in small numbers.

Numbers included with a previous cancer or a cancer demonstrated at first round, were insufficient to draw a firm conclusion. The impression was, however, that those having had a cancer had the same risk for a new primary as those who had not had a previous cancer. This group was not the goal for the current study, and we do not discuss this further.

Penetrance observed for *BRCA2 *mutation carriers was similar to *BRCA1 *founder mutations. From the literature, we had expected lower. Both King et al. 2003 and Antoniou et al 2003 did report increasing penetrance of BRCA mutations today compared to previous generations. Our findings may support their notion. If this were to be true, it may explain some of the controversies in retrospective studies in different countries and ethnic groups. This adds to variation in penetrances and methodological problems, as discussed above, to explain controversies between retrospective reports.

Prospectively observed survival of patients with *BRCA1 *mutations and with breast or ovarian cancer, is 50% or less in 10 years [13,15]. It follows that the risk of dying from breast or ovarian cancer is at least half the annual incidence rates of contracting cancer reported here. Prospects for better treatment modalities and prevention strategies are outside the aims of the current report and are not discussed here.

In conclusion, we have confirmed high penetrances of *BRCA1 *and *BRCA2 *mutations in a prospective series. The annual incidence rates presented are empirical observations which may be used for genetic counseling of healthy women a with stop, frameshift or large deletion/insertion mutation in the *BRCA1 *or *BRCA2 *genes. In the given age groups, our results may be considered annual risk of breast or ovarian cancer in the next 6 years to come. In addition, our findings may give reason to examine further whether or not rare *BRCA1 *mutations may be rare because they have lower fitness than the most frequent *BRCA1 *mutations.

## Competing interests

The authors declare that they have no competing interests.

## Authors' contributions

The study was designed by PM. Data were collected by by PM, LM, LFE, TL, CJ, JA and AV. IT systems were constructed and data for current analysis extracted by PM and NC. The manuscript was written and approved by all authors.
